# Immunogenicity, safety, and efficacy of rurioctocog alfa pegol in previously untreated patients with severe hemophilia A

**DOI:** 10.1016/j.rpth.2026.106939

**Published:** 2026-09-03

**Authors:** Robert F. Sidonio, Eric S. Mullins, Flora Peyvandi, Anthony K.C. Chan, Oleksandra Stasyshyn, Seoh Leng Yeoh, Darintr Sosothikul, Ali Bulent Antmen, Samer S. El-Kamary, Joan Gu, Srilatha Tangada

**Affiliations:** 1 Aflac Cancer and Blood Disorders Center, Emory University, Atlanta, Georgia, USA; 2 Division of Hematology, Cancer and Blood Diseases Institute, Cincinnati Children’s Hospital Medical Center and University of Cincinnati College of Medicine, Cincinnati, Ohio, USA; 3 Fondazione IRCCS Ca’ Granda Ospedale Maggiore Policlinico, Angelo Bianchi Bonomi Hemophilia, Milan, Italy; 4 Thrombosis Centre and Fondazione Luigi Villa, Milan, Italy; 5 McMaster Children’s Hospital, Hamilton, Ontario, Canada; 6 Academy of Medical Sciences of Ukraine, Lviv, Ukraine; 7 Department of Pediatrics, Hospital Pulau Pinang, George Town, Malaysia; 8 Department of Pediatrics, Faculty of Medicine, Chulalongkorn University, Bangkok, Thailand; 9 Center of Excellence in Pediatric Hematology/Oncology, Faculty of Medicine, Chulalongkorn University, Bangkok, Thailand; 10 Department of Pediatric Hematology and Oncology, Acibadem Adana Hospital, Adana, Türkiye; 11 Takeda Development Center Americas, Inc, Cambridge, Massachusetts, USA; 12 Takeda Development Center Americas, Inc, Lexington, Massachusetts, USA

**Keywords:** antibodies, child, factor VIII, hemophilia A, neutralizing, prospective studies

## Abstract

**Background:**

In severe hemophilia A, factor VIII (FVIII) replacement is essential, but FVIII inhibitor development can be serious.

**Objectives:**

This phase 3, prospective, uncontrolled, open-label, multicenter study (NCT02615691) investigated the immunogenicity, efficacy, and safety of rurioctocog alfa pegol for severe hemophilia A in previously untreated patients.

**Methods:**

Patients received intravenous prophylaxis (25-50 IU/kg, up to 80 IU/kg, ≥1 dose weekly) and/or on-demand therapy (10-50 IU/kg, up to 80 IU/kg depending on bleed severity). The primary endpoint was FVIII inhibitor incidence (≥0.6 BU/mL [Nijmegen modification of Bethesda assay]) in the full analysis set (patients with ≥100 exposure-days [EDs] or who developed an inhibitor). We present the final data.

**Results:**

Of 146 enrolled patients, 120 received ≥1 dose; all were male, and 55.8% had a family history of hemophilia A. Median (range) age was 9.6 (1.2-60.0) months. In the full analysis set (*N* = 100), 11 patients developed FVIII inhibitors, and 89 had ≥100 EDs without developing inhibitors (incidence [95% CI], 0.110 [0.056-0.188]). In *post hoc* analyses, 11 patients developed inhibitors, and 101 and 96 had ≥20 and ≥50 EDs, respectively, without developing inhibitors (incidence [95% CI], 0.098 [0.050-0.169] and 0.103 [0.052-0.177], respectively). Annualized bleeding rates (95% CI) were 1.7 (1.2-2.4), 1.0 (0.7-1.3), and 2.3 (1.7-3.2) for once-weekly prophylaxis, >1× weekly prophylaxis, and on-demand therapy, respectively. Overall, 109 of 120 (90.8%) patients had 745 adverse events; serious adverse events occurred in 47 (39.2%) patients.

**Conclusion:**

These data demonstrate a low incidence of FVIII inhibitors among previously untreated patients receiving rurioctocog alfa pegol for severe hemophilia A. Efficacy and safety results were similar to prior reports in previously treated patients.

## Graphical abstract

**Figure:**
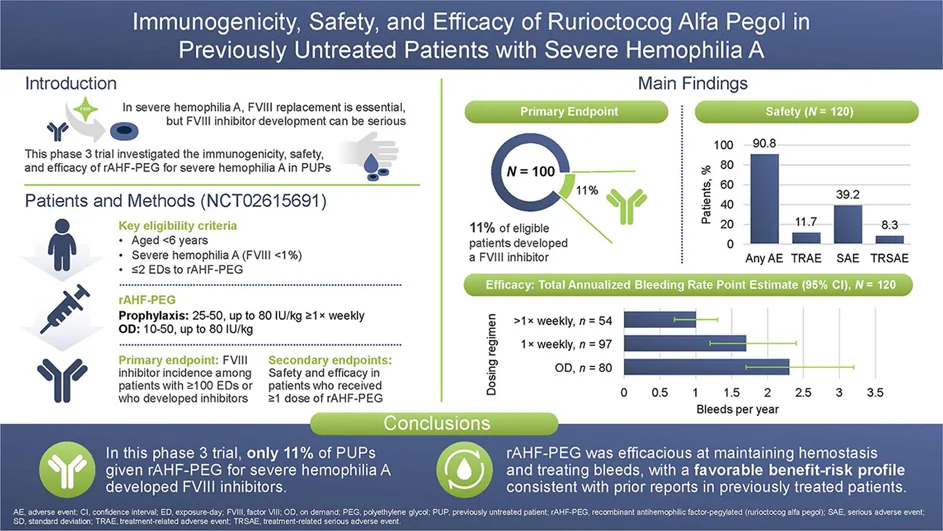

## 1 Introduction

Hemophilia A is a congenital, recessive, X-linked disorder in which factor VIII (FVIII) deficiency leads to frequent, acute, and prolonged bleeding events [1,2]. For severe hemophilia A (FVIII activity <1%), the current standard of care includes prophylactic FVIII replacement or nonfactor therapies such as emicizumab [[2], [3], [4], [5]]. Previously untreated patients (PUPs) with severe hemophilia A are at risk of developing FVIII inhibitors [6], which are associated with substantially increased morbidity and health care costs [[7], [8], [9]]. FVIII inhibitors develop in approximately 20% to 35% of PUPs with severe hemophilia A, usually occurring soon (<20 exposure-days [EDs]) after the initiation of exogenous FVIII administration [6,[10], [11], [12], [13]].

FVIII replacement therapies with an extended half-life may reduce treatment burden, as they allow the frequency of administration to be reduced without compromising efficacy [14]. Rurioctocog alfa pegol (ADYNOVATE [US]/ADYNOVI [Europe]; Baxalta US Inc, a Takeda company, and Baxalta Innovations GmbH, a Takeda company) is a recombinant FVIII (rFVIII) product with an extended half-life [15,16]. It is manufactured by covalently bonding 20-kDa polyethylene glycol (PEG) chains, consisting of a branched PEG derivative with two 10-kDa PEG chains, to octocog alfa (ADVATE; Baxalta US Inc, a Takeda company) [16,17]. Although PEGylation can improve the pharmacokinetic properties of drugs, such modifications can alter their immunogenic properties [[18], [19], [20]]. It is therefore essential to evaluate the immunogenicity of novel PEGylated products such as rurioctocog alfa pegol in patients (especially PUPs) with hemophilia A.

Interim results from this phase 3 open-label study in PUPs with severe hemophilia A suggested that rurioctocog alfa pegol was associated with a lower incidence of FVIII inhibitors compared with octocog alfa (19% vs 27.8% for patients with ≥50 EDs and ≥75 EDs, respectively) [13,21], while maintaining efficacy and a similar safety profile to studies in previously treated patients [22]. Here, we present the final data from this study, after 120 PUPs with severe hemophilia A received ≥1 dose of rurioctocog alfa pegol.

## 2 Methods

### 2.1 Study design, patient population, and treatment

The design of this phase 3, prospective, uncontrolled, open-label, nonrandomized multicenter study (NCT02615691) has been published previously [13]. Briefly, the study was conducted between November 2015 and October 2024. Patients aged <6 years with severe hemophilia A and ≤2 EDs to rurioctocog alfa pegol, octocog alfa, or plasma transfusion were eligible for inclusion. Patients with detectable FVIII inhibitory antibodies at screening or a history of FVIII inhibitory antibodies before screening (≥0.6 BU/mL) were excluded.

Patients received intravenous rurioctocog alfa pegol as prophylaxis (25-50 IU/kg, up to 80 IU/kg at the investigator’s discretion, at least once weekly) and/or on-demand therapy (10-50 IU/kg, up to 80 IU/kg depending on bleed severity). Treatment assignment, dosing, and prophylactic frequency were based on individual clinical characteristics and investigator discretion. Participants younger than 3 years with <2 joint bleeds could receive either on-demand therapy or prophylaxis. Participants who commenced on-demand treatment were required to transition to prophylaxis by 3 years of age or if they experienced 2 joint bleeds. Participants ≥3 years of age or with ≥2 joint bleeds received prophylaxis. Patients who developed either high-titer FVIII inhibitors (>5.0 BU/mL) or low-titer inhibitors (≥0.6 to ≤5.0 BU/mL) plus poorly controlled bleeding, despite increasing FVIII doses and/or bypassing agents during the study, were eligible for immune tolerance induction (ITI). When inhibitor development was confirmed by the central laboratory, ITI could be commenced at the discretion of the investigator. Dosing for ITI therapy ranged between 50 IU/kg 3 times per week (low dose) and 100 to 200 IU/kg daily (high dose) at the investigator’s discretion.

The study was conducted in compliance with the International Council for Harmonisation Good Clinical Practice guidelines and the Declaration of Helsinki, as well as other applicable national and local ethical and legal requirements. Written informed consent was obtained from the parent or legal guardian for each participant. The protocol, final approved consent/assent document, relevant supporting material, and all patient recruitment information were reviewed and approved by the relevant institutional review boards before study initiation.

### 2.2 Outcome measures

The primary outcome was incidence of FVIII inhibitor development (≥0.6 BU/mL using the Nijmegen modification of the Bethesda assay) [23], based on the full analysis set (all patients with ≥100 EDs or who developed a confirmed FVIII inhibitor). Development of FVIII inhibitors was assessed at all study visits, determined by a central laboratory, and confirmed by a second blood sample drawn within 2 weeks of site notification of an inhibitor. To ensure timely availability of FVIII inhibitor results, the clinical management of the patient for ITI could be based on results generated at the local laboratory. Rates of FVIII inhibitor development at 20 and 50 EDs were evaluated in a *post hoc* analysis.

Secondary objectives included safety and efficacy. Safety was assessed as immunogenicity, which included binding IgG and IgM antibodies to FVIII, PEG-FVIII, and PEG, assessed at all study visits. Adverse events (AEs) and serious AEs (SAEs) were assessed throughout the study. Efficacy was assessed as annualized bleeding rate (ABR) for prophylactic and on-demand treatment. Overall hemostatic efficacy ratings after 24 hours and at bleed resolution were also assessed. Consumption was assessed by the number of rurioctocog alfa pegol infusions per bleeding episode and weight-adjusted consumption of rurioctocog alfa pegol per month, per year, and per event.

Clinical laboratory assessments conducted throughout the trial included FVIII activity assays, hematology, and clinical chemistry. Genetics and human leukocyte antigen-genotype were assessed at screening, and CD4 count was assessed at screening and annually thereafter. Cell pellets were retained for epigenetic analyses. FVIII trough levels were used to guide dosing during perioperative management and after successful ITI treatment where relevant.

### 2.3 Statistical analysis

Demographic and baseline characteristics were summarized for the safety analysis set, comprising all patients who received ≥1 dose of rurioctocog alfa pegol.

Continuous variables were reported as mean (SD), and categorical variables as number and percentage. FVIII gene variants were classified as low or high risk based on the traditional risk classification for inhibitor formation [24].

The incidence of FVIII inhibitor development was assessed using the Clopper-Pearson exact 95% CI for the proportion of patients who developed inhibitors during the study. ITI was classified as a success {inhibitor titer persistently <0.6 BU/mL, FVIII incremental recovery ≥66% of baseline, and FVIII half-life of ≥6 hours (according to Hay et al. [25])}, partial success (meeting 2 of these 3 criteria after 33 months), or failure (not meeting the success or partial success criteria after 33 months, or achieving <20% inhibitor titer reduction). In this analysis, tolerization refers to ITI success or partial success.

ABR was analyzed by point and interval estimates derived by SAS procedure GLIMMIX (SAS Institute Inc, 2020, SAS/STAT 15.2 User’s Guide) from a negative binomial model with the treatment regimen as a covariate and the logarithm of the duration of the regimen as an offset. Descriptive statistics were given for rurioctocog alfa pegol consumption.

Missing data were not imputed, except if body weight was unavailable (in which case, the last available value was carried forward to calculate the weight-adjusted consumption) or if the date of onset of an AE was completely missing (in this case, it was imputed as the date of the first receipt of the study drug). Otherwise, all analyses were performed using nonmissing data.

## 3 Results

### 3.1 Patient population

In total, 146 patients were enrolled from 55 study sites in 19 countries. Of these patients, 120 received ≥1 dose of rurioctocog alfa pegol and were included in the safety analysis set (Figure 1). Of these 120 patients, 40 received prophylaxis only, 8 received on-demand treatment only, and 72 received both prophylaxis and on-demand treatment. All patients were male, and the majority (55.8%) had a family history of hemophilia A (Table 1). Median (range) age at informed consent was 9.6 (1.2-60.0) months. One hundred patients had ≥100 EDs or developed a confirmed FVIII inhibitor and qualified for evaluation of the primary endpoint.

**Figure 1:**
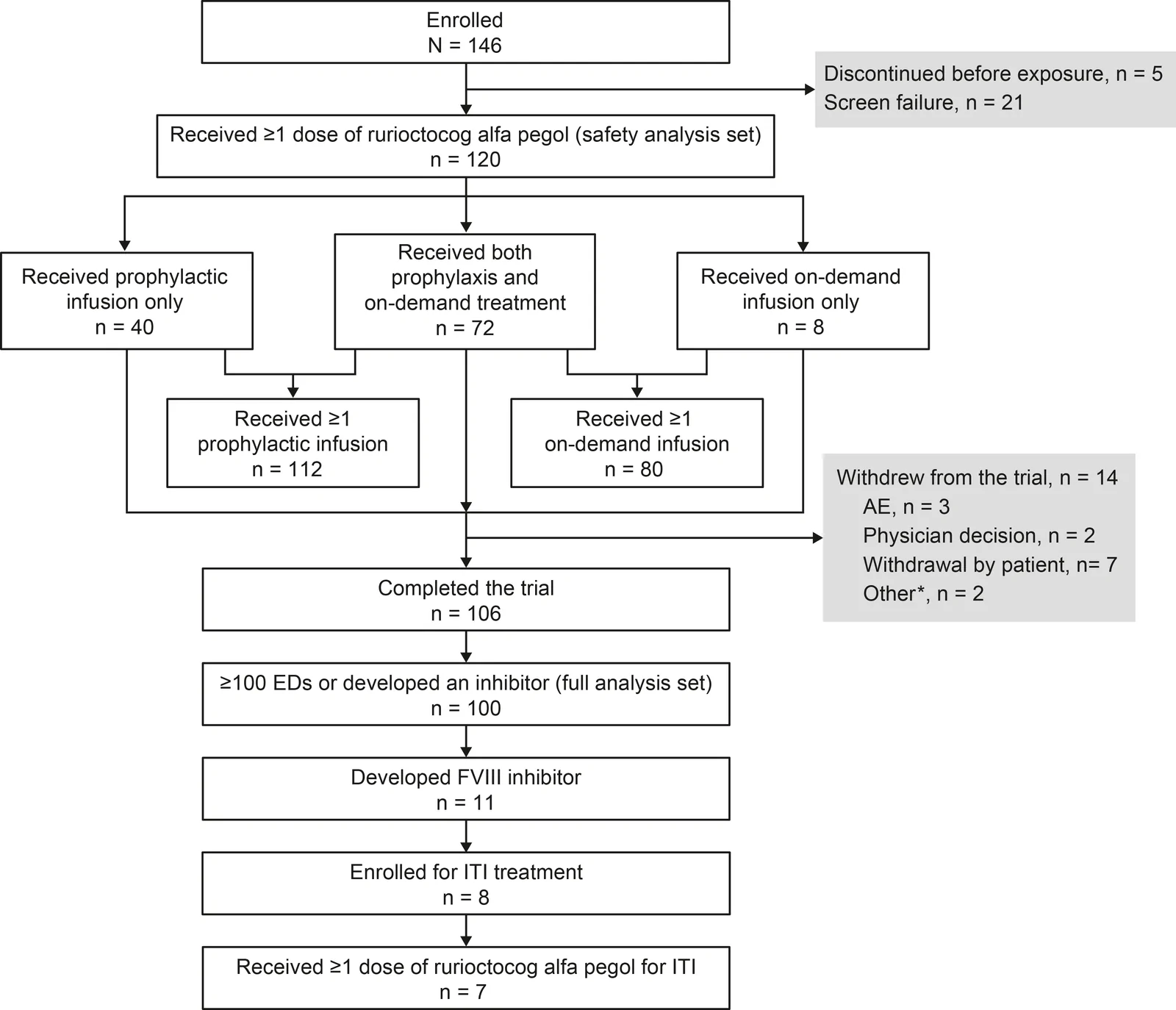
Patient disposition. ∗Other includes prohibited concomitant medication for treatment of the bleed and using a drug other than the study drug during the bleeding. AE, adverse event; ED, exposure-day; FVIII, factor VIII; ITI, immune tolerance induction.

**Table 1:** Baseline demographic and disease characteristics (*N* = 120).

Variable	Value
Age at informed consent (mo), mean (SD)^a^	10.8 (8.6)
Median (range) (mo)	9.6 (1.2-60.0)
Male, *n* (%)	120 (100)
Race, *n* (%)	
Asian	45 (37.5)
Black or African American	7 (5.8)
White	61 (50.8)
Other/multiple	7 (5.8)
Country, *n* (%)	
United States	32 (26.7)
Malaysia	24 (20.0)
Türkiye	16 (13.3)
Thailand	12 (10.0)
United Kingdom	9 (7.5)
Ukraine	5 (4.2)
Belgium	3 (2.5)
India	3 (2.5)
Italy	3 (2.5)
Other^b^	13 (10.8)
Family history of hemophilia A, *n* (%)^c^	67 (55.8)
Grandfather	15 (12.5)
Father^d^	1 (0.8)
Uncle	28 (23.3)
Brother	25 (20.8)
Other relatives^e^	29 (24.2)
Family history of FVIII inhibitors, *n* (%)	6 (5.0)
No. of exposure-days before screening, *n* (%)	
0	73 (60.8)
1	22 (18.3)
2	25 (20.8)
Exposure-days before screening, mean (SD)^f^	0.6 (0.8)
Prior treatment with FVIII products, *n* (%)^g^	47 (39.2)
Rurioctocog alfa pegol	0
Octocog alfa	36 (30.0)
Fresh frozen plasma	13 (10.8)
Weight (kg), mean (SD)	9.8 (2.9)
Height (cm), mean (SD)	74.5 (9.8)
BMI (kg/m^2^), mean (SD)	17.4 (2.3)
Hemophilia-associated gene variants, *n* (%)	
High risk	79 (65.8)
Intron 22 inversion	56 (46.7)
Large structural change	1 (0.8)
Large deletion	4 (3.3)
Nonsense mutation	7 (5.8)
Small duplication frameshift^h^	2 (1.7)
Small deletion/nonsense	2 (1.7)
Small deletion frameshift^h^	4 (3.3)
Small insertion frameshift	1 (0.8)
Intron 1 inversion	2 (1.7)
Low risk	33 (27.5)
Small duplication frameshift^h^	8 (6.7)
Missense mutation	18 (15.0)
Small deletion frameshift^h^	3 (2.5)
Frameshift	1 (0.8)
Small deletion/in-frame/splice site	1 (0.8)
Splice site	1 (0.8)
In-frame	1 (0.8)
Missing	8 (6.7)

BMI, body mass index; FVIII, factor VIII.

a Age at informed consent as reported on the electronic case report form, converted to months.

b Other includes Canada, France, Norway, Singapore, South Korea (each *n* = 2), Hong Kong, Spain, and Taiwan (each *n* = 1).

c Patients could have had more than 1 relative with hemophilia A.

d Although a patient cannot inherit a gene defect on the X chromosome (as with hemophilia A) from his father, 1 patient did have a father with hemophilia A (and either had a spontaneous mutation of the X chromosome or inherited a defective X chromosome from his mother).

e Other relatives included cousin/mother’s cousin/first, second, or third cousin (*n* = 10), grandchildren (*n* = 1), granduncle/great uncle (*n* = 4), grandmother’s grandfather (*n* = 1), mother (*n* = 1), and mother’s cousin (*n* = 1).

f Exposure-days before screening was defined as the unique calendar days that the patient received octocog alfa, fresh frozen plasma, or rurioctocog alfa pegol before screening.

g Two patients received prior treatment with octocog alfa and fresh frozen plasma. These patients were counted in both treatment categories.

h Small deletions/insertions/duplications could be classified as either high or low risk depending on location and the type of sequence affected; for example, small deletions/insertions within a stretch of adenine repeats were classified as low risk.

Using the traditional risk classification for inhibitor formation [24], 79 (65.8%) patients had high-risk FVIII gene variants and 33 (27.5%) patients had low-risk FVIII gene variants (Table 1). The most common FVIII gene variants were intron 22 inversion (56 patients; 46.7%) and missense variants (18 patients; 15.0%).

### 3.2 Immunogenicity

In the primary outcome assessment, of the 100 patients in the full analysis set, 11 (11.0%) developed inhibitory antibodies to FVIII and 89 had ≥100 EDs without developing an inhibitor, equating to an inhibitor incidence of 0.110 (95% CI, 0.056-0.188). In the *post hoc* analysis, among the 112 patients with ≥20 EDs or who developed an inhibitor, 11 developed an inhibitor and 101 had ≥20 EDs without developing an inhibitor (inhibitor incidence, 0.098 [95% CI, 0.050-0.169]). Of the 107 patients with ≥50 EDs or who developed an inhibitor, 11 developed inhibitors and 96 had ≥50 EDs without developing an inhibitor, for an incidence of 0.103 (95% CI, 0.052-0.177).

The mean (SD) number of EDs until confirmed inhibitor development was 7.6 (4.0); no inhibitors developed after 20 EDs (Figure 2). High-titer inhibitors (>5.0 BU/mL) were present in 6 of the 11 patients (across these 6 patients, the range in highest titer was 7.3-2004.1 BU/mL). Low-titer inhibitors (≥0.6 to ≤5.0 BU/mL) were present in 5 of the 11 patients (across these 5 patients, the range in highest titer was 1.1-4 BU/mL). Of the 11 patients, 10 had high-risk FVIII gene variants and 1 patient had a low-risk FVIII gene variant. Eight of these patients were White and 3 were Asian. Patient age ranged from 3 months to 4 years. Six patients had a family history of hemophilia A, and 1 patient had a family history of FVIII inhibitors. Of those who developed FVIII inhibitors, 4 had received prior treatment with octocog alfa or fresh frozen plasma. No patients had received prior treatment with rurioctocog alfa pegol. At the time of confirmed inhibitor development, 3 were receiving on-demand treatment, and 8 were receiving rurioctocog alfa pegol prophylaxis.

**Figure 2:**
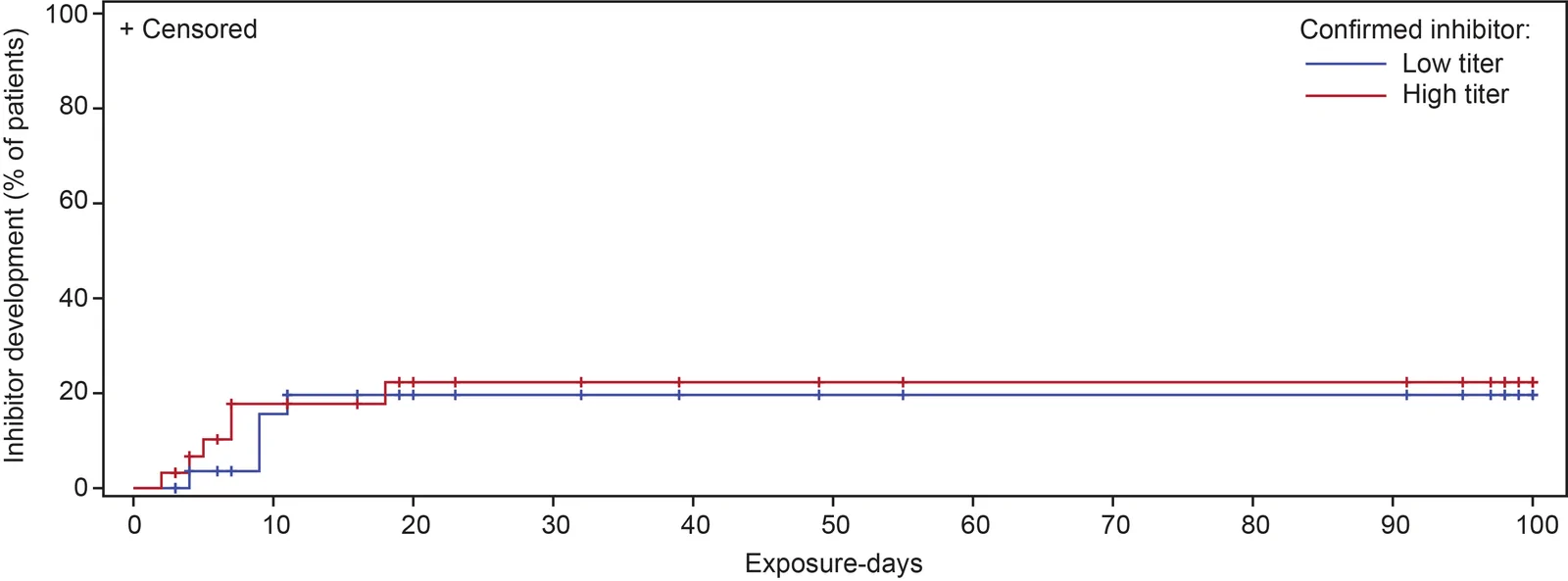
Kaplan-Meier analysis of time to confirmed low- and high-titer inhibitors.

Of the 11 patients who developed FVIII inhibitors to rurioctocog alfa pegol during the study, 8 were enrolled to receive ITI, and 7 received ≥1 dose of rurioctocog alfa pegol as ITI (Figure 1). After ITI treatment, 6 of the 7 patients were considered tolerized, and 1 had ITI failure.

### 3.3 Efficacy

The 120 patients included in the safety analysis set had 15,001 EDs over a total observation period of 297.1 years. The mean (SD) total EDs to rurioctocog alfa pegol was 124.3 (103.4).

Of these patients, 112 received prophylaxis with rurioctocog alfa pegol (12,785 EDs; observation period, 286.3 years; mean [SD] EDs, 114.2 [96.2]). A total of 107 patients received rurioctocog alfa pegol to treat bleeds (952 EDs; observation period, 282.1 years; mean [SD] EDs, 8.9 [8.3]). Seventy-two patients switched from on-demand treatment to prophylaxis. Patients switched treatment at a median age of 2.0 years, after a mean (SD) of 9.8 (7.5) EDs.

The total ABR (95% CI) using the negative binomial model was 1.7 (1.2-2.4) for prophylaxis once weekly (*n* = 97), 1.0 (0.7-1.3) for prophylaxis more than once per week (*n* = 54), and 2.3 (1.7-3.2) for on-demand therapy (*n* = 80). For prophylaxis once weekly, prophylaxis more than once per week, and on-demand therapy, respective annualized joint bleeding rates (95% CI) were 0.3 (0.2-0.5), 0.2 (0.1-0.4), and 0.6 (0.3-1.0), and annualized spontaneous/unknown causality bleeding rates were 1.3 (0.7-2.4), 0.4 (0.2-0.8), and 1.9 (1.0-3.9). Zero bleeds were reported for 5 patients who received treatment for 200 to 1172 days (99-126 EDs).

During the first part of the study (before FVIII inhibitor detection and the start of ITI therapy), 1059 bleeds occurred (prophylaxis once weekly, 436 bleeds; prophylaxis more than once per week, 119 bleeds; on-demand, 489 bleeds). Overall, 775 bleeds (occurring in 106 patients) were treated with rurioctocog alfa pegol. The remaining 284 bleeds were not treated, as they were mostly superficial skin bleeds. Of the total treated bleeds, 169 (21.8%) occurring in 50 patients were spontaneous, 484 (62.5%) in 99 patients were related to injury, and 121 (15.6%) in 44 patients were of unknown causality. The numbers of bleeds of minor, moderate, and major severity were 386, 340, and 47, occurring in 87, 87, and 24 patients, respectively. The number of joint bleeds was 151 in 60 patients.

Most bleeds occurred >96 hours after treatment (Supplementary Figure). Hemostatic efficacy was assessed in 109 participants. Hemostatic efficacy of treatment, as rated by patients or their caregivers, is shown in Table 2. Where efficacy was reported, it was mostly rated as “excellent” or “good” after 24 hours (316 of 342 [92.4%]) and at resolution (492 of 517 [95.2%]) for all bleeds.

**Table 2:** Hemostatic efficacy of rurioctocog alfa pegol in prophylactic treatment and control of bleeds.

Category	All bleeds^a^		
	On-demand (*n* = 379)	Prophylaxis (*n* = 396)	Total (*N* = 775)
Efficacy rating after 24 h, *n* (%)	*n* = 158	*n* = 184	*n* = 342
Excellent	76 (48.1)	102 (55.4)	178 (52.0)
Good	67 (42.4)	71 (38.6)	138 (40.4)
Fair	13 (8.2)	10 (5.4)	23 (6.7)
None	2 (1.3)	1 (0.5)	3 (0.9)
Efficacy rating at bleeding resolution, *n* (%)	*n* = 264	*n* = 253	*n* = 517
Excellent	166 (62.9)	166 (65.6)	332 (64.2)
Good	83 (31.4)	77 (30.4)	160 (30.9)
Fair	12 (4.5)	9 (3.6)	21 (4.1)
None	3 (1.1)	1 (0.4)	4 (0.8)

Data are reported for the safety analysis set (*N* = 120).

a Some bleeds were not rated because of a misunderstanding of case report form completion guidelines; if a bleed resolved within a few hours after 1 infusion, it was mistakenly thought that a rating was not required.

Of 775 bleeding events, most (614 [79.2%]) were treated with a single infusion of rurioctocog alfa pegol. Two infusions were required for 87 (11.2%) bleeds, 3 infusions for 37 (4.8%) bleeds, and 4 infusions for 5 (0.6%) bleeds; 11 (1.4%) bleeds required >4 infusions. The mean (SD) average dose per infusion to treat a bleed was 40.4 (14.6) IU/kg. For prophylaxis, mean (SD) monthly infusions and dose per month were 4.9 (1.9) and 221.6 (85.9) IU/kg, respectively (Table 3).

**Table 3:** Weight-adjusted consumption and number of infusions for patients receiving rurioctocog alfa pegol prophylaxis (*N* = 112).

Variable	Value
No. of infusions per month, mean (SD)	4.9 (1.9)
Prophylactic dose per month (IU/kg), mean (SD)	221.6 (85.9)
Dose per prophylactic infusion (IU/kg), mean (SD)	45.5 (7.8)
No. of infusions required for bleed resolution, mean (SD)	1.3 (1.0)
Average dose for bleed resolution (IU/kg), mean (SD)	63.9 (44.8)

### 3.4 Safety

Overall, 109 of the 120 (90.8%) patients who received ≥1 dose of rurioctocog alfa pegol experienced a total of 745 AEs in the first part of the study (Table 4). SAEs were reported in 47 (39.2%) patients, including 11 patients each with 1 SAE of FVIII inhibitor development. Of the 11 SAEs of FVIII inhibitor development, 10 were related to rurioctocog alfa pegol prophylaxis (1 treatment-related AE of FVIII inhibitor development that occurred during the main study was not included in this analysis because it was reported during the ITI portion of the study).

**Table 4:** AEs occurring during or after first dose of rurioctocog alfa pegol.

AEs	Patients who received ≥1 dose of rurioctocog alfa pegol	
	Patients, *n* (%) (*N* = 120)	Events, *n*
Any AE	109 (90.8)	745
Frequently reported AEs^a^		
Pyrexia	43 (35.8)	95
Upper respiratory tract infection	34 (28.3)	69
Cough	23 (19.2)	35
Nasopharyngitis	20 (16.7)	37
Viral infection	15 (12.5)	20
Diarrhea	12 (10.0)	15
Gastroenteritis	12 (10.0)	12
Treatment-related AEs	14 (11.7)	17^b^
FVIII inhibitor development	10 (8.3)^c^	10^c^
Hypersensitivity	2 (1.7)	2
Eyelid rash	1 (0.8)	1
Vomiting	1 (0.8)	1
Blood alkaline phosphatase increased^d^	1 (0.8)	1
Cough	1 (0.8)	1
Rash	1 (0.8)	1
Catheter-related AEs	0	0
Thrombotic AEs	0	0
Any SAE	47 (39.2)	70
SAEs occurring in ≥2 patients		
FVIII inhibitor development^e^	11 (9.2)	11
Hemarthrosis	5 (4.2)	5
Pyrexia	4 (3.3)	5
Muscle hematoma	3 (2.5)	3
Muscle hemorrhage	2 (1.7)	2
Road traffic accident	2 (1.7)	2
Gastroenteritis	2 (1.7)	2
Pneumonia	2 (1.7)	2
Treatment-related SAEs	10 (8.3)	10
FVIII inhibitor development^e^	10 (8.3)	10
AEs leading to treatment discontinuation	5 (4.2)^f^	15
AEs leading to study discontinuation	3 (2.5)	9
AEs leading to death	0	0
AEs related to study procedure	6 (5.0)	7
SAEs related to study procedure	3 (2.5)	3

AE, adverse event; FVIII, factor VIII; SAE, serious adverse event.

a Reported in ≥10% of patients who received ≥1 dose of rurioctocog alfa pegol in the safety analysis set.

b Investigator-assessed value is 17 events; the sponsor-assessed value is 16 events^d^.

c One treatment-related AE of FVIII inhibitor development that occurred during the main study is not included in this analysis because it was reported during the immune tolerance induction portion of the study.

d One AE of blood alkaline phosphatase increased was assessed as related to treatment by the investigator, but assessed as not related to treatment by the sponsor, as the abnormal value was related to a laboratory error.

e One SAE of FVIII inhibitor development was not related to rurioctocog alfa pegol and instead was related to treatment with octocog alfa before infusion of rurioctocog alfa pegol.

f Three patients discontinued because of FVIII inhibitor development and 2 because of drug hypersensitivity.

Two (1.7%) participants had a hypersensitivity reaction deemed related to rurioctocog alfa pegol. No thrombotic AEs or catheter-related AEs related to rurioctocog alfa pegol were reported. Six (5.0%) patients experienced 7 AEs related to the study procedure, and 3 (2.5%) patients experienced 3 SAEs related to the trial procedure (Table 4).

## 4 Discussion

This phase 3 trial was the first to investigate the immunogenicity, safety, and efficacy of rurioctocog alfa pegol for the treatment of PUPs with severe hemophilia A [13]. Data from 120 PUPs demonstrate a low incidence of FVIII inhibitor development coupled with a similar safety profile and similar efficacy compared with prior reports in previously treated patients.

Among PUPs with ≥100 EDs to rurioctocog alfa pegol or who developed an inhibitor, 11% developed inhibitors, lower than prior reports for PUPs receiving other extended half-life rFVIII products, such as the 30.0% reported for turoctocog alfa pegol (*n* = 70) [26], 27.2% for efmoroctocog alfa (*n* = 103) [9], and 26.7% for the standard half-life rFVIII simoctocog alfa (*n* = 105) [27]. Similarly, among patients with ≥20 EDs to rurioctocog alfa pegol or who developed an inhibitor, 9.8% developed inhibitors compared with 20.8% for an interim analysis of the above study with simoctocog alfa (*n* = 66) [28]. Among the 107 patients with ≥50 EDs or who developed an inhibitor, 10.3% developed inhibitors in this study, lower than prior clinical trials for PUPs receiving other FVIII products: 26.8% with ≥50 EDs to plasma-derived FVIII (PUPs and minimally treated patients) [11], 44.5% with ≥50 EDs to rFVIII (pooled data for octocog alfa, octocog alfa [bhk] and moroctocog alfa among PUPs and minimally treated patients) [11], 43.1% with ≥50 EDs to turoctocog alfa [29], 54.5% with 50 EDs to lonoctocog alfa [30], and 22.8% with ≥50 EDs to clotting factor concentrate [31]. Reports based on other exposure thresholds include an inhibitor incidence of 27.8% for patients with ≥75 EDs to octocog alfa [21] and 31.1% for patients with ≥10 EDs to efmoroctocog alfa [32]. These clinical trial data are consistent with real-world data on PUPs and previously treated patients from the European Haemophilia Safety Surveillance System, which reported 9 cases of first inhibitor development to efmoroctocog alfa in 2024, along with 6 cases of first inhibitor development to standard half-life rFVIII products, including 1 event for octocog alfa [33]. Although 390 patients received rurioctocog alfa pegol in 2024, there were no reports of first or recurrent inhibitors to rurioctocog alfa pegol [33].

This difference in immunogenicity between rurioctocog alfa pegol and other FVIII products could be because rurioctocog alfa pegol consists of a full-length B-domain, whereas other extended half-life FVIII products use a B-domain–deleted, truncated, and genetically modified rFVIII [17,34,35]. Protein modeling studies and biophysical data suggest that the B-domain could shield key FVIII epitopes, potentially reducing immune interference and preventing premature activation [36,37]. Another reason could be differences in the PEGylation process. With the addition of two 10-kDa PEG branches, rurioctocog alfa pegol uses the smallest PEG moiety among PEGylated FVIII products, which may reduce the risk of intracellular accumulation, resulting from the lower uptake by phagocytic cells, development of antibodies against PEG, and hypersensitivity reactions [17,34,38,39]. In addition, attachment of the PEG moieties to the flexible B-domain may enable the PEG molecules to shield the immunogenic epitopes on the drug surface, resulting in lower recognition by the immune system, and thereby decreasing the production of neutralizing antibodies and adverse immune reactions [37,40,41]. The reduced immunogenicity compared with unmodified full-length rFVIII, octocog alfa, is particularly notable because both products are composed of the same parent protein and presented and formulated with identical excipients [15,42]. This suggests that the PEGylation process led to a reduction in immunogenicity. Another possible explanation for the differences in inhibitor rates could be population characteristics. Approximately two-thirds (65.8%) of the patients in this study had high-risk FVIII mutations, similar to previous studies (75.7%, 79.6%, and 82.0% [includes minimally treated patients]) [9,21,32]. In addition, >80% of PUPs and minimally treated patients in the Survey of Inhibitors in Plasma-Product Exposed Toddlers (SIPPET) study recorded null mutations that were associated with an increased incidence of inhibitors (hazard ratio, 1.81; 95% CI, 0.83-3.95) [11].

Despite differences in inhibitor development rates between rurioctocog alfa pegol and other FVIII products, time to inhibitor development in this final analysis of the current study (mean, 7.6 days) was similar to that in the interim analysis of our study (mean, 8.0 days) [13] and in previous studies of rFVIII products in PUPs and minimally treated patients (mean, 10.9-14.2 days [11,29]; median, 8-13 days) [11,21,27,32]. In our study, all inhibitors occurred within 20 EDs, similar to studies of other rFVIII products, in which all instances of inhibitor development occurred within 24 EDs [29], 26 EDs [21], and 39 EDs [10]. Although not the focus of the present study, this lower inhibitor development rate with rurioctocog alfa pegol may be expected to have a positive clinical impact by reducing mortality rates, hospitalizations, and treatment costs typically associated with persistent inhibitors [2,7,8]. This could be assessed in future studies.

The efficacy of rurioctocog alfa pegol prophylaxis is supported by the total ABRs during prophylactic treatment (1.7 and 1.0 for weekly and more than once per week, respectively). ABRs of 1.4 to 4.4 have been reported for other hemophilia A treatment options; however, direct comparisons cannot be drawn between trials because of the differing methods of ABR calculation and differences in the target population [26,32,[43], [44], [45]]. As expected, the ABR in the present study was numerically lowest in patients receiving rurioctocog alfa pegol prophylaxis more than once per week (1.0) and highest in those receiving on-demand therapy (2.3). The interim analysis of our study reported an ABR of 3.2 for both on-demand and prophylactic therapy [13], which may have been an artifact of the shorter follow-up period. Most bleeds (62.5%) were related to injury, which is higher than in a prior phase 2/3 study of rurioctocog alfa pegol in previously treated patients (40.1%) [46]. This may be due to the age of participants in the current trial being <6 years, compared with 12 to 65 years in the previous study. This lower ABR among patients receiving regular prophylaxis vs on-demand therapy in the current trial is consistent with prior studies in which prophylaxis was associated with a reduced incidence of bleeding, joint hemorrhage, and arthropathy compared with on-demand therapy [47,48], and supports guideline recommendations to provide prophylaxis instead of on-demand therapy for the management of hemophilia [2].

Although international guidelines for the management of hemophilia recommend prophylaxis over on-demand treatment [2], on-demand treatment is permitted as per the product label in countries including the United States [16], and real-world data show that both on-demand and prophylactic regimens are used in clinical practice [49]. This study permitted on-demand treatment for individuals <3 years of age with <2 joint bleeds; however, patients were required to initiate prophylaxis by the age of 3 years or if they experienced 2 joint bleeds, whichever occurred first. Inclusion of both on-demand and prophylactic treatment regimens is important to obtain accurate immunogenicity data reflective of real-world clinical practice, and is in line with prior studies assessing immunogenicity of FVIII products in PUPs [9,11,26]. During the study, 72 patients switched from on-demand treatment to prophylaxis, supporting improved outcomes in line with guideline recommendations.

Most patients experienced AEs, with 39.2% experiencing an SAE. This is similar to prior reports in previously treated pediatric, adolescent, and adult patients with hemophilia A [46,[50], [51], [52]]. There were 2 hypersensitivity reactions and no thrombotic or catheter-related AEs. No new safety concerns were identified. These safety and efficacy data are important despite the availability of non-FVIII therapies, as FVIII replacement can be required even in patients receiving non-FVIII prophylaxis, during surgery, life-threatening bleeds, or significant trauma [[53], [54], [55]].

Strengths of this study include a diverse patient population (enrolled from 19 countries), increasing the generalizability of the results. Limitations include the uncontrolled open-label design. In addition, limiting the study to patients with ≤2 prior EDs might have reduced inhibitor risk estimates by excluding patients who are highly susceptible to inhibitor formation.

The final results from this study show that, among PUPs receiving rurioctocog alfa pegol for severe hemophilia A, the rate of FVIII inhibitor development was low compared with the expected rate based on data from other standard and extended half-life FVIII products. Rurioctocog alfa pegol was efficacious at maintaining hemostasis and treating bleeds, with a favorable benefit-risk profile that is consistent with prior reports in previously treated patients.
