# Supplementary material for: Immunogenicity, safety, and efficacy of rurioctocog alfa pegol in previously untreated patients with severe hemophilia A
Source: Res Pract Thromb Haemost. 2026 Sep 3;10(6):106939. doi: 10.1016/j.rpth.2026.106939 (PMC13641043; doi:10.1016/j.rpth.2026.106939)
Supplement: Supplementary file 1 [file mmc1.docx]

**Supplementary Materials**

**List of investigators***

| **Investigator** | **Affiliation** |
| --- | --- |
| Albayrak Canan | Division of Pediatric Hematology and Oncology, Ondokuz Mayis University Faculty of Medicine, Samsun, Türkiye |
| Ali Bulent Antmen | Department of Pediatric Hematology and Oncology, Acibadem Adana Hospital, Adana, Türkiye |
| Amy Knott | University Hospitals Bristol and Weston NHS Foundation Trust, Bristol, UK |
| Angela C. Weyand | Division of Pediatric Hematology Oncology, Department of Pediatrics University of Michigan Medical School, Ann Arbor, MI |
| Anthony K.C. Chan | McMaster Children's Hospital, Hamilton, ON, Canada |
| Catherine McGuinn | Weill Cornell Medicine, New York, NY |
| Christoph Male | Department of Pediatrics, Medical University of Vienna, Vienna, Austria |
| Ampaiwan Chuansumrit | Department of Pediatrics, Faculty of Medicine Ramathibodi Hospital, Mahidol University, Bangkok, Thailand |
| Darintr Sosothikul | Department of Pediatrics, Faculty of Medicine, Chulalongkorn University, Bangkok, Thailand;  Center of Excellence in Pediatric Hematology/Oncology, Faculty of Medicine, Chulalongkorn University, Bangkok, Thailand |
| Elaine Eyster | Penn State Hershey Medical Center, Hershey, PA |
| Eric S. Mullins | Division of Hematology, Cancer and Blood Diseases Institute, Cincinnati Children’s Hospital Medical Center and University of Cincinnati College of Medicine, Cincinnati, OH |
| Flora Peyvandi | Fondazione IRCCS Ca’ Granda Ospedale Maggiore Policlinico, Angelo Bianchi Bonomi Hemophilia, Milan, Italy; Thrombosis Centre and Fondazione Luigi Villa, Milan, Italy |
| Godfrey Chi-Fung Chan | Department of Paediatrics and Adolescent Medicine, LKS Faculty of Medicine, The University of Hong Kong, Hong Kong |
| Heidi Glosli | Department of Pediatric Research and Norwegian Centre for Rare Diseases, Division of Pediatric and Adolescent Medicine, Oslo University Hospital, Oslo, Norway |
| Jayanthi Alamelu | Evelina London Children’s Hospital, London, UK |
| Jessica Meznarich | University of Utah/Primary Children's Hospital, Division of Pediatric Hematology/Oncology/BMT, Salt Lake City, UT |
| John D. Grainger | Royal Manchester Children's Hospital, Manchester, UK; University of Manchester, Manchester, UK |
| Jonathan Ducore | Pediatrics-Hematology/Oncology Director- Hemostasis and Thrombosis Center, UC Davis, Sacramento, CA |
| Joyce Ching Mei Lam | KK Women's and Children's Hospital, Singapore |
| Komvilaisak Patcharee | Department of Pediatrics, Faculty of Medicine, Khon Kaen University, Khon Kaen, Thailand |
| Phu Quoc Le | Hemato-oncology unit, Hôpital Universitaire Des Enfants Reine Fabiola, Brussels, Belgium |
| Maria Teresa Alvarez Roman | Hematology Department, La Paz University Hospital-IdiPaz, Universidad Autónoma de Madrid, Madrid, Spain |
| Rungrote Natesirinilkul | Faculty of Medicine, Chiang Mai University, Chiang Mai, Thailand |
| Oleksandra Stasyshyn | Academy of Medical Sciences of Ukraine, Lviv, Ukraine |
| Bunchoo Pongtanakul | Division of Hematology and Oncology, Department of Pediatrics, Faculty of Medicine Siriraj Hospital, Mahidol University, Bangkok, Thailand |
| Prakas Kumar Madal | NRS Medical College, West Bengal, India |
| Shashikant Janardan Apte | Sahyadri Speciality Hospital, Pune, India |
| Robert F. Sidonio Jr | Aflac Cancer and Blood Disorders Center, Emory University, Atlanta, GA |
| Seoh Leng Yeoh | Department of Pediatrics, Hospital Pulau Pinang, George Town, Malaysia |
| Susen Sophie | Univ. Lille, Inserm, Department of Hemostasis and Transfusion CHU Lille, Institut Pasteur de Lille, Lille, France |
| Tung Wynn | Division of Pediatric Hematology and Oncology  University of Florida, Gainesville, FL |
| Veerle Labarque | Department of Pediatric Hematology-Oncology and Stem Cell Transplantation, Ghent University Hospital, Ghent University, Ghent, Belgium |
| Jiaan-Der Wang | Center for Rare Disease and Hemophilia, Taichung Veterans General Hospital, Taichung, Taiwan |
| Young-Shil Park | Department of Pediatrics, Kyung Hee University Hospital at Gangdong, Kyung Hee University College of Medicine, Seoul, Republic of Korea |
| Zulfikar Bulent | Department of Paediatric Haematology/Oncology, Istanbul University Oncology Institute, Istanbul, Türkiye |

*Only investigators who provided permission to be included are listed.

**Data monitoring committee members***

| **Name** | **Affiliation** |
| --- | --- |
| Rolf Ljung | Dept. of Clinical Sciences Lund – Paediatrics, Lund University, Lund, Sweden |
| Sandrine Meunier | Hospices Civils de Lyon - French Reference Center for Haemophilia, Clinical Haemostasis Unit, Hopital Louis Pradel, Lyon, France |

*Only members who provided permission to be included are listed.

**Supplemental Figure. Number of bleeding episodes following treatment.**
